# Proteomic analysis of secretagogue-stimulated neutrophils implicates a role for actin and actin-interacting proteins in Rac2-mediated granule exocytosis

**DOI:** 10.1186/1477-5956-9-70

**Published:** 2011-11-14

**Authors:** Gary Eitzen, Andrea N Lo, Troy Mitchell, John D Kim, Danny V Chao, Paige Lacy

**Affiliations:** 1 Department of Cell Biology, University of Alberta, Edmonton, AB, Canada; 2 Pulmonary Research Group, Department of Medicine, University of Alberta, Edmonton, AB, Canada

**Keywords:** neutrophil, degranulation, exocytosis, Rac2, GTPase, actin, coronin

## Abstract

**Background:**

Neutrophils are abundant leukocytes that play a primary role in defence against pathogens. Neutrophils enter sites of infection where they eliminate pathogens via phagocytosis and the release of antimicrobial mediators via degranulation. Rho GTPases, particularly Rac2, play a key role in neutrophil degranulation. The purpose of this study was to identify Rac2-dependent changes in protein abundance in stimulated neutrophils.

**Methods:**

We performed a proteomic analysis on secretagogue-stimulated bone marrow neutrophils that were isolated from wild-type and Rac2^-/- ^mice. Protein abundance was analyzed by 2-dimensional SDS-PAGE of fluorescently labelled samples which allowed the detection ~3500 proteins.

**Results:**

We identified 22 proteins that showed significant changes in abundance after secretagogue-stimulation of wild-type neutrophils, which did not occur in neutrophils isolated from Rac2^-/- ^mice. As expected, the abundance of several granule proteins was reduced in wild-type cells; this did not occur in Rac2^-/- ^neutrophils which confirms the requirement for Rac2 in degranulation. We also found changes in abundance of many actin remodelling proteins including coronin-1A, β-actin and the F-actin capping protein, (CapZ-β). Coronin-1A showed elevated levels of several isoforms after stimulation of neutrophils from wild-type, but not from Rac2^-/- ^mice. These isoforms were immunoreactive with anti-phospho-threonine antibodies, suggesting that neutrophil stimulation triggers a Rac2-dependent kinase cascade that results in the phosphorylation of coronin-1A.

**Conclusion:**

The control of Rac2-mediated degranulation in neutrophils likely functions through actin remodelling via activation of several actin-binding proteins. We found coronin-1A to be a novel downstream effector protein of this pathway that is threonine phosphorylated in response to secretagogue stimulation.

## Background

Neutrophils are the most abundant circulating blood leukocyte, which play a crucial role in innate immunity. Neutrophils rapidly accumulate at sites of infection and act to contain and destroy invading microbial pathogens. These cells possess a potent arsenal of cytotoxic proteins such as oxidants, proteinases and antimicrobial peptides to facilitate pathogen clearance [1,2]. They also release immunoregulatory cytokines and chemokines that recruit and activate other inflammatory cells.

Neutrophil activation results in the release of inflammatory mediators and up-regulation of cell surface adhesion molecules through a process known as degranulation. Degranulation involves graded exocytosis of at least 4 different granule types: primary (azurophilic), secondary (specific), and tertiary granules, along with secretory vesicles [3,4]. The exocytosis of primary granules is highly regulated since they contain highly reactive proteolytic enzymes, cationic proteins, as well as myeloperoxidase which generates oxidative substances. The regulation of primary granule exocytosis likely involves cytoskeletal remodelling since these granules associate with actin and actin interacting proteins [5]. The current model is that actin is required to mobilize granules to the cell surface for fusion with the plasma membrane during receptor-induced secretion [6].

The molecular mechanism that activates neutrophil exocytosis is poorly understood. We, and others, have shown that Rac2, a member of the Rho GTPase subfamily of *ras*-related GTPases that stimulates the formation of F-actin in neutrophils, is essential for neutrophil primary granule exocytosis [7-10]. Gene deletion of Rac2 in mice also leads to a loss of chemotactic ability in peripheral blood and bone marrow neutrophils, along with reduced superoxide production in response to the bacterially derived tripeptide formyl-Met-Leu-Phe (fMLF), tumour necrosis factor (TNF) or phorbol myristate acetate (PMA) [11]. Kinase activity is altered in Rac2-deficient neutrophils, as the phosphorylation of p38 MAP kinase and ERK1/2 activity was partially diminished in response to PMA and opsonised zymosan. [11,12]. However, little is known regarding the signaling mechanisms associated with Rac2 in response to secretagogue stimulation.

Neutrophil degranulation in response to fMLF is a rapid process, taking less than 15 min, which makes this cellular event highly amenable to protein analysis techniques. Any protein modification during this time is likely to be post-translational since this time is insufficient for *de novo *synthesis. We predicted that using this short time of stimulation will increase the probability of detecting post-translation modifications, however, changes in abundance due to proteolysis and secretion would also be detected.

In this study, neutrophils from wild-type and Rac2^-/- ^mice were stimulated with cytochalasin B (CB)/fMLF, a potent secretagogue combination, and differences in protein abundance were identified using a unique system of fluorescent protein labelling and detection after 2D gel electrophoresis. Differences in protein abundance between samples were determined via quantitative fluorescence detection. Spots were mapped on multiple gels and computational analysis was used to match spot maps and identify changes in spot intensities. These experiments were repeated several times, allowing statistical calculations on inter-gel variations in spot intensities. We were interested in proteins (i.e. spots) that changed significantly in abundance between wild-type and Rac2^-/- ^samples. Several proteins of interest were identified by mass spectrometry (MS) and compared to a database of known protein sequences and masses. We predict that granule proteins will decrease in abundance in wild-type neutrophils following stimulation by CB/fMLF, while other proteins will increase in abundance, particularly if they are involved in promoting granule exocytosis. Furthermore, changes in protein abundance will be altered in Rac2^-/- ^neutrophils compared with wild-type cells if they are part of the regulatory pathway downstream of Rac2. The purpose of this study was to generate a global profile of protein abundance changes in stimulated neutrophils, and to specifically identify those changes that do not occur in neutrophils isolated from Rac2^-/- ^mice.

## Results

### Generation of neutrophil proteomic sample sets

**Figure 1:**
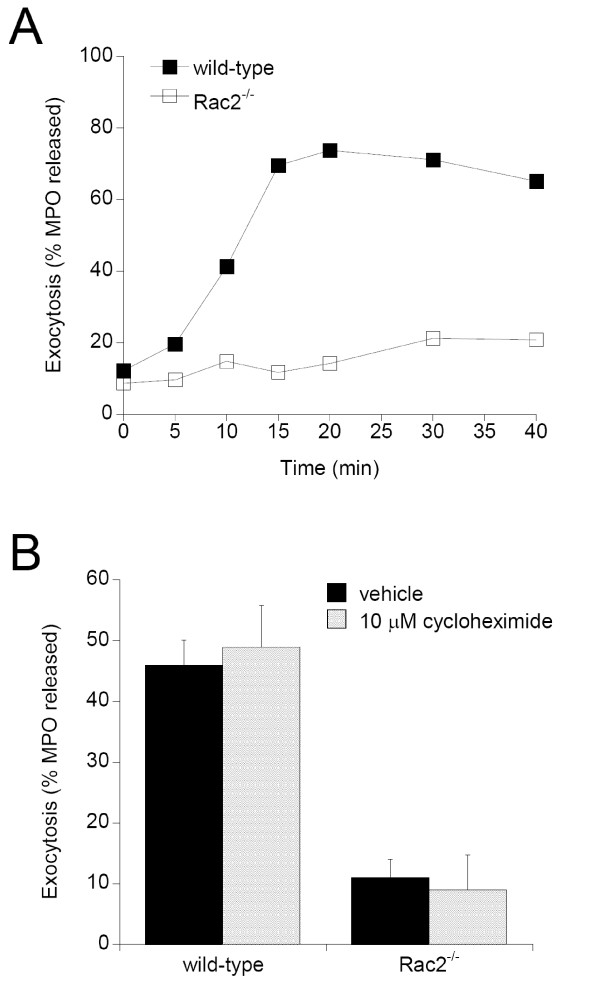
**Kinetic analysis of neutrophil exocytosis**. Neutrophils were isolated from bone marrow of wild-type and Rac2^-/- ^mice. 1 × 10^6 ^cells were incubated with 5 μM CB for 5 min followed by 5 μM fMLF. At the indicated times samples were taken and cell-free supernatants were analyzed for MPO, an enzyme contained in primary granules. Exocytosis was measure as the % MPO release, calculated as a ratio of total cellular MPO. **A**. Shown is a representative result from a typical degranulation assays. **B**. Pre-incubation of BMN with 10 μM cycloheximide for 1 hr at 37°C prior to CB/fMLF stimulation does not affect degranulation (n = 3).

**Figure 2:**
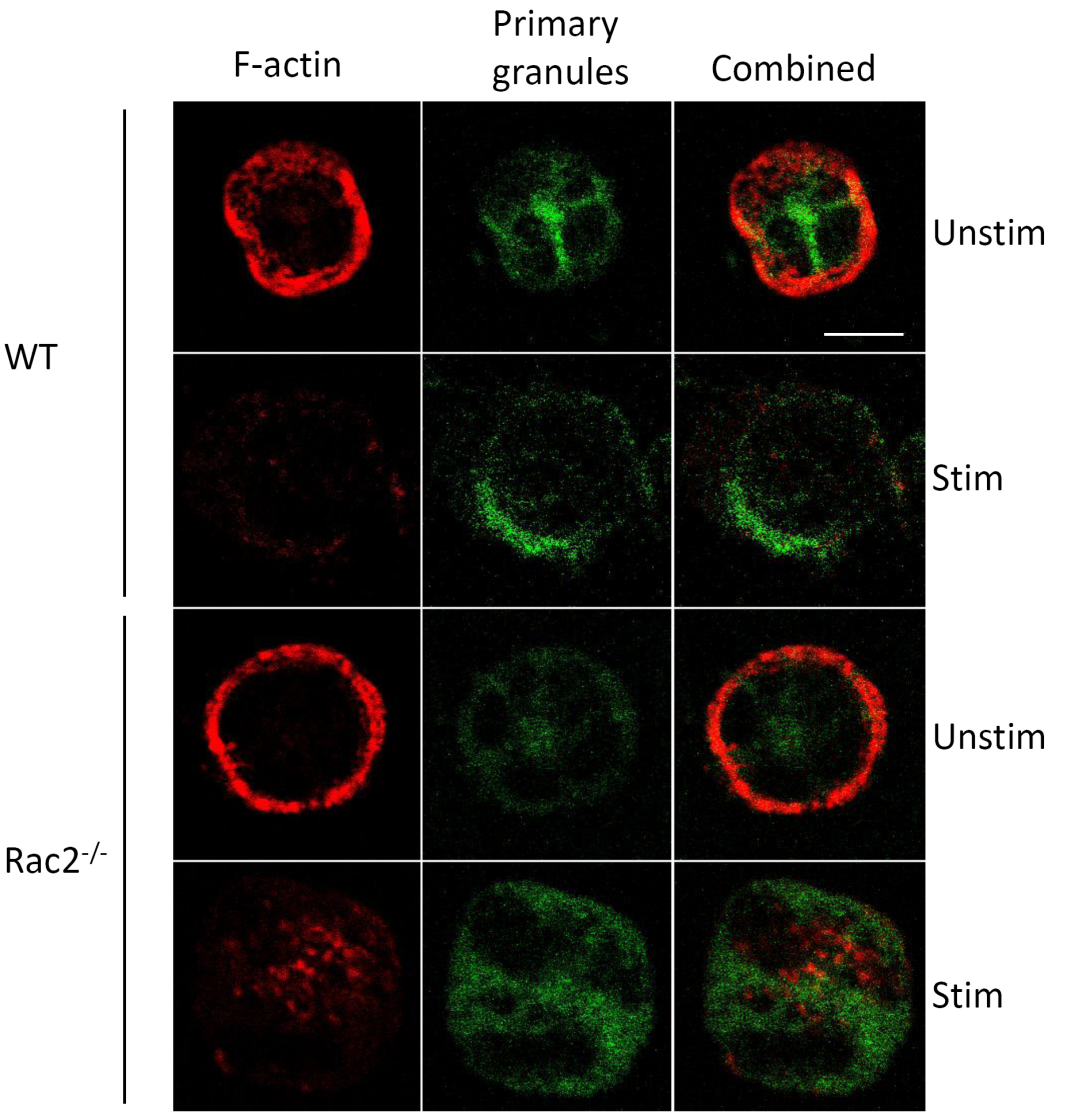
**Microscopic analysis of granules and F-actin**. Cytospins of BMNs freshly prepared from wild-type and Rac2^-/- ^mice showing F-actin structures stained with phalloidin (*red*) and primary granules stained with CD63 (*green*). Cortical actin staining is more intense in unstimulated Rac2^-/- ^cells compared to wild-type cells (*unstim*). The F-actin ring structure is dispersed in both cells following 15 min of CB/fMLF stimulation (*stim*). 60x/1.4 numerical aperture objective; bar = 5 μm.

Rac2 is a hematopoietic-specific GTPase which is required for the activation of neutrophil immune cell functions, including granule exocytosis and activation of the NADPH oxidase complex [7,10]. Here, we performed a proteomic screen to characterize the Rac2-dependent neutrophil response to secretagogue stimulation, which activates exocytosis (degranulation). Our goal was to identify proteins which may be post-translationally modified in response to stimulation. Rac2-dependent changes can be identified by comparing samples prepared from wild-type versus Rac2^-/- ^mice. Three independent experiments were performed, whereby bone marrow neutrophils (BMN) were isolated from two wild-type and two Rac2^-/- ^mice. Cells were incubated with vehicle (unstimulated) or the secretagogue CB/fMLF (stimulated) for 15 min at 37°C. A 15 min stimulation was selected as the ideal time-point for comparative analyses because this is the time required to obtain maximal degranulation in mouse neutrophils (Figure 1A). We have previously shown that Rac2 activation (the formation of Rac2-GTP) is sustained for 15 min when using CB/fMLF as the secretagogue [10]. As well, this would be insufficient time to allow *de novo *protein synthesis, which focuses our proteomic screen on Rac2-dependent post-translational modifications, proteolysis or secretion. Pre-incubation of neutrophils with the translation inhibitor cycloheximide did not affect degranulation, confirming that *de novo *protein synthesis is not required for degranulation (Figure 1B). Examination of BMN by microscopy revealed no obvious morphological differences in cell shape and structure other than more intense cortical actin in unstimulated Rac2^-/- ^cells (Figure 2, *unstim*). Stimulation resulted in the redistribution of azurophilic CD63^+ ^granule staining which accumulated at the cell periphery in wild-type cells but not in Rac2^-/- ^cells (Figure 2, *stim*).

The proteomes of wild-type and Rac2^-/- ^neutrophils were analyzed by subjecting whole cell lysates to two-dimensional fluorescence-difference gel electrophoresis (2D-DiGE) for spot-by-spot comparison of abundance [13,14]. Proteins from each condition were labelled with Cy3 and Cy5 and a pooled sample of all conditions was labelled with Cy2 to allow gel-to-gel normalization (*see *Additional File 1: Figure S1, for a schematic representation of sample preparation). Cy3 and Cy5 labelled samples were combined with the Cy2 normalization sample and run on 2D gels which were used to quantify protein abundance by spot densitometry using the Ettan DiGE system (GE Healthcare). The experiment was run three times with each condition represented six times for a total of 12 gels, to facilitate robust statistical analyses. The DeCyder differential in-gel analysis (DIA) software module was used to processes individual gel images; this computational analysis performed spot detection and quantification (abundance), in-gel normalization, background subtraction and gel artefact removal. The data from all the gels was then transferred to the biological variance analysis (BVA) software module; this computational analysis performed statistical processing of inter-gel and inter-experiment variation of protein abundance. From this analysis, we selected 22 spots of interest which all showed greater than 1.5-fold change (increase or decrease) in abundance when comparing wild-type unstimulated versus stimulated samples, and wild-type versus Rac2^-/- ^stimulated samples.

### 2D gel spot map analysis

**Figure 3:**
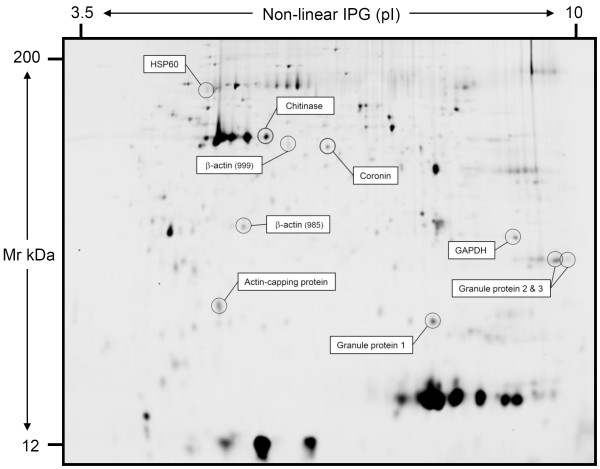
**Representative image of 2-dimensional proteomics gel**. Protein samples were isolated from BMN and differentially labelled with fluorophores (see Methods). Shown is the pooled sample from wild-type BMN labelled with Cy2. Proteins identified by quantitative differential analysis and mass spectrometry are indicated on the gel.

22 spots showing highly relevant changes (*p *< 0.01) in abundance were chosen for identification by mass spectrometry. Fluorescent gel spot maps were matched to master Coomassie-stained gels, and spots were picked and analyzed by LC/MS/MS. Ten of 22 spots were identified with greater than 15% coverage in wild-type and Rac2^-/- ^neutrophils (Figure 3; *see *Additional File 2: Table S1, for MS data). None of the spots chosen for sequencing were Rac2, indicating that this approach was not sensitive enough to detect the loss of Rac2 expression in Rac2^-/- ^BMN. Indeed, no regulatory or signaling molecules such as kinases or phosphatases could be detected.

**Figure 4:**
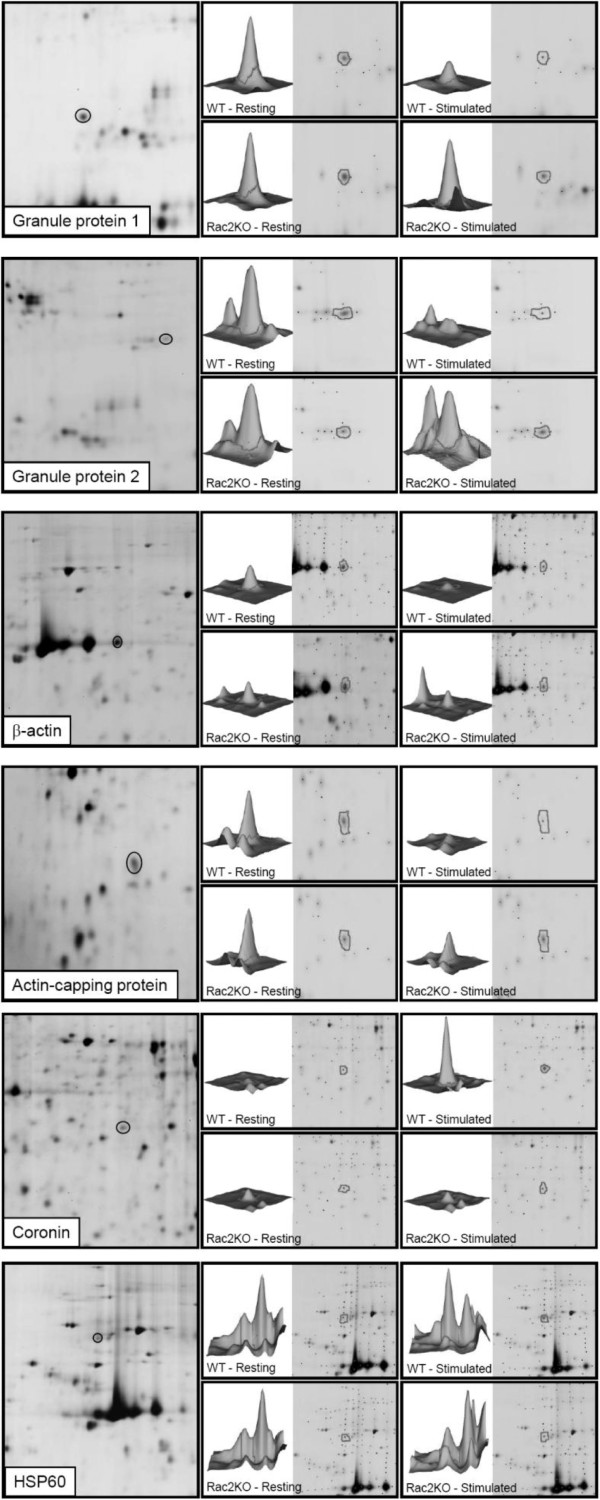
**Magnified regions of 2D gels corresponding to identified proteins**. Spots of interest from 2D gels (*left panel*) were quantified in four different samples corresponding to unstimulated and stimulated wild-type and Rac2^-/- ^BMN (*right four panels*). The intensity of spots is also shown as 3D histograms.

**Figure 5:**
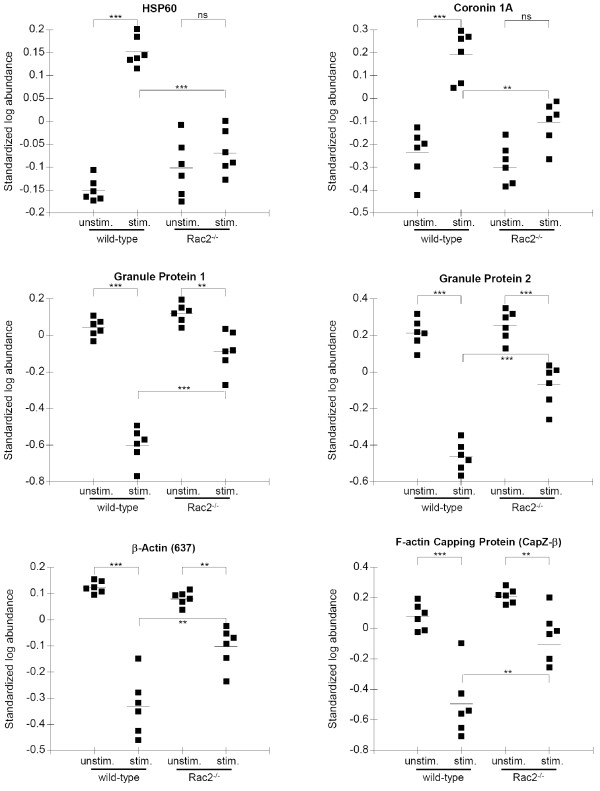
**Scatter plots of standardized log abundance of identified proteins**. Abundance of spots was determined from fluorescently labelled samples of BMN run on 2D gels. Standardization of spots was to a pooled sample run on all six gels. The statistical significance is shown for paired comparisons (n = 6 for each condition). **, *p *< 0.01; ***, *p *< 0.001; ns, *p *> 0.05 (not significant).

Abundance data was derived from spot mapping and densitometry using the DeCyder software DIA module. The accuracy of the mapping procedure was examined which showed that spot maps were uniform and included only the primary area of the spot for densitometry (Figure 4). Table 1 shows the changes in abundance between wild-type unstimulated and stimulated samples of the identified proteins with negative ratios indicating reduced protein abundance (e.g. due to protein secretion), while positive ratios indicate an increase in protein abundance (e.g. due to protein modification). Likewise, Table 2 shows changes in abundance between Rac2^-/- ^unstimulated and stimulated samples. Table 3 shows the changes in abundance between wild-type stimulated and Rac2^-/- ^stimulated neutrophils. Abundance data of six identified proteins is graphically represented by scatter plot in Figure 5, which highlights the changes in protein abundance that occurred in wild-type neutrophils, and that did not occur to the same extent in Rac2^-/- ^neutrophils.

**Table 1:** Proteins identified by mass spectrometry and their differences in spot abundance compared between wild-type unstimulated and stimulated BMN.

Identity	Master Number	NIH protein database accession number	Wild-type Unstimulated (abundance)	Wild-type Stimulated (abundance)	Ratio	p-value (*t *test)
Coronin	691	NP_034028	0.0673	0.192	2.85	5.3 × 10^-4^
GAPDH	425	AAU89484	0.0603	0.128	2.14	6.1 × 10^-7^
HSP60	382	NP_034607	0.0524	0.105	2.01	3.2 × 10^-5^
Chitinase	1018	NP_075675	3.66	0.803	-4.56	2.8 × 10^-7^
Granule protein 1	965	BAB26414	0.284	0.0728	-3.90	2.8 × 10^-5^
β-actin	985	ABL01512	0.265	0.0898	-2.95	3.0 × 10^-4^
Granule protein 2	969	NP_032720	0.196	0.0598	-3.28	6.6 × 10^-5^
β-actin	637	ABL01512	0.0841	0.0306	-2.75	8.7 × 10^-6^
F-actin capping protein (CapZ-β)	999	NP_033928	0.162	0.0478	-3.39	8.7 × 10^-4^
Granule protein 3	970	EDL09016	0.0552	0.0108	-5.11	9.8 × 10^-6^

HSP60, heat shock protein; GAPDH, glyceraldehyde 3-phosphate dehydrogenase; NIH, National Institutes of Health.

**Table 2:** Proteins identified by mass spectrometry and their differences in spot abundance compared between Rac2^-/^^- ^unstimulated and stimulated BMN.

Identity	Master Number	NIH protein database accession number	Rac2^-/- ^Unstimulated (abundance)	Rac2^-/- ^Stimulated (abundance)	Ratio	p-value (*t *test)
Coronin	691	NP_034028	0.0655	0.0990	1.51	0.13
HSP60	382	NP_034607	0.0572	0.0652	1.14	0.14
GAPDH	425	AAU89484	0.0696	0.0731	1.05	0.56
Granule protein 1	965	BAB26414	0.244	0.151	-1.62	3.1 × 10^-2^
β-actin	637	ABL01512	0.0729	0.0517	-1.41	1.5 × 10^-2^
F-actin capping protein (CapZ-β)	999	NP_033928	0.161	0.0951	-1.69	5.9 × 10^-3^
Granule protein 2	969	NP_032720	0.158	0.0891	-1.79	2.2 × 10^-3^
Chitinase	1018	NP_075675	4.39	2.23	-1.97	4.9 × 10^-4^
Granule protein 3	970	EDL09016	0.0539	0.0258	-2.09	1.6 × 10^-2^
β-actin	985	ABL01512	0.342	0.151	-2.25	4.4 × 10^-3^

HSP60, heat shock protein; GAPDH, glyceraldehyde 3-phosphate dehydrogenase; NIH, National Institutes of Health.

**Table 3:** Proteins identified by mass spectrometry and their differences in spot abundance compared between wild-type stimulated and Rac2^-/^^- ^stimulated BMN.

Identity	Master Number	NIH protein database accession number	Wild-type Stimulated (abundance)	Rac2^-/- ^Stimulated (abundance)	Ratio	p-value (*t *test)
Granule protein 3	970	EDL09016	0.0108	0.0258	2.39	4.1 × 10^-4^
Granule protein 2	969	NP_032720	0.0598	0.0891	1.49	0.18
F-actin capping protein (CapZ-β)	999	NP_033928	0.0478	0.0951	1.99	6.1 × 10^-2^
β-actin	637	ABL01512	0.0306	0.0517	1.69	1.1 × 10^-2^
Granule protein 1	965	BAB26414	0.0728	0.151	2.07	2.6 × 10^-3^
β-actin	985	ABL01512	0.0898	0.152	1.69	7.3 × 10^-2^
Chitinase	1018	NP_075675	0.803	2.23	2.77	9.0 × 10^-5^
GAPDH	425	AAU89484	0.128	0.0731	-1.75	7.9 × 10^-5^
HSP60	382	NP_034607	0.105	0.0652	-1.61	6.2 × 10^-4^
Coronin	691	NP_034028	0.192	0.0990	-1.94	4.0 × 10^-3^

HSP60, heat shock protein; GAPDH, glyceraldehyde 3-phosphate dehydrogenase; NIH, National Institutes of Health.

As expected, the abundance of granule proteins decreased in wild-type stimulated neutrophils, which was expected since these proteins would normally be depleted due to granule exocytosis (Figure 5; Tables 1). By comparison, granule proteins did not decrease in neutrophils from Rac2^-/- ^mice (Figure 5; Table 3), which is consistent with the need for Rac2 in exocytosis [7]. A decrease in the abundance of cytoskeletal proteins, β-actin and F-actin capping protein subunit beta (CapZ-β) was also observed in wild-type BMNs. CapZ-β is an actin interacting protein that, along with CapZ-α, regulates the availability of free barbed-ends to grow new actin filaments. CapZ-α subunits were identified in a proteomic analysis of proteins associated with neutrophil primary granules [15]. Significant changes in GAPDH and chitinase were also observed in wild-type samples when stimulated, however, their changes in abundance in Rac2^-/- ^samples were similar to wild-type (Figure 5, Tables 1 and 3).

Two proteins, coronin-1A and HSP60, were identified that showed an increase in abundance in wild-type cells (Figures 4 and 5). In contrast, Rac2^-/- ^neutrophils did not showed a significant increase in HSP60 and coronin-1A abundance following secretagogue stimulation. Coronins are F-actin-binding proteins that are involved in reorganization of the actin cytoskeleton, in concert with the Arp2/3 complex and ADF/cofilin [16]. Coronin-1A is specifically involved in the regulation of leukocyte signaling events [17,18]. HSP60 is a cytosolic protein that was recently shown to be important in neutrophil chemotaxis and enhancement of superoxide release and degranulation, leading to increased inflammation [19]. The increase in coronin-1A and HSP60 abundance in wild-type BMNs is likely due to post-translational modifications, since the time course of the experiment (15 min) is insufficient to allow *de novo *protein synthesis [13]. As well, inhibition of protein synthesis with cycloheximide did not affect degranulation (Figure 1B).

### Analysis of changes in actin and coronin-1A abundance

**Figure 6:**
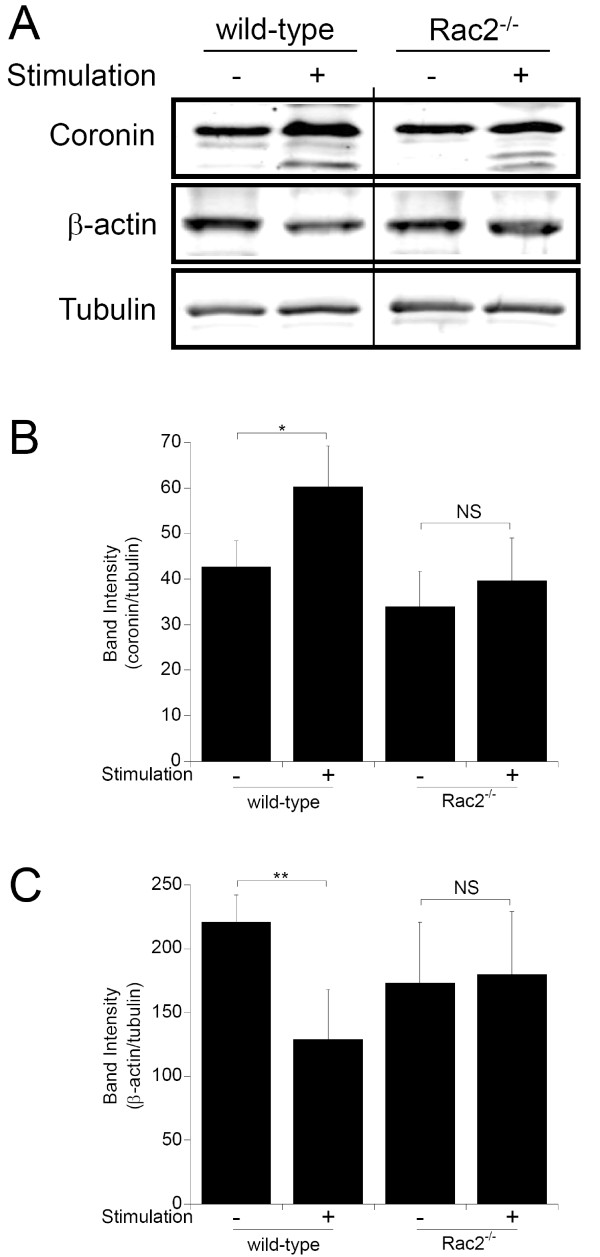
**Immunoblot for actin and coronin in BMN samples**. Cell lysates were prepared from BMN isolated from wild-type and Rac2^-/- ^mice. Cells were either unstimulated or stimulated for 15 min with CB/fMLF. **A**. Lysates were probed for coronin-1A, β-actin and β-tubulin. **B and C**. Quantification of coronin-1A (***B***) and β-actin (***C***) band intensities measured from three samples, normalized to β-tubulin for each sample (n = 3). *, *p *< 0.05; **, *p *< 0.01; ns, *p *> 0.05 (not significant).

**Figure 7:**
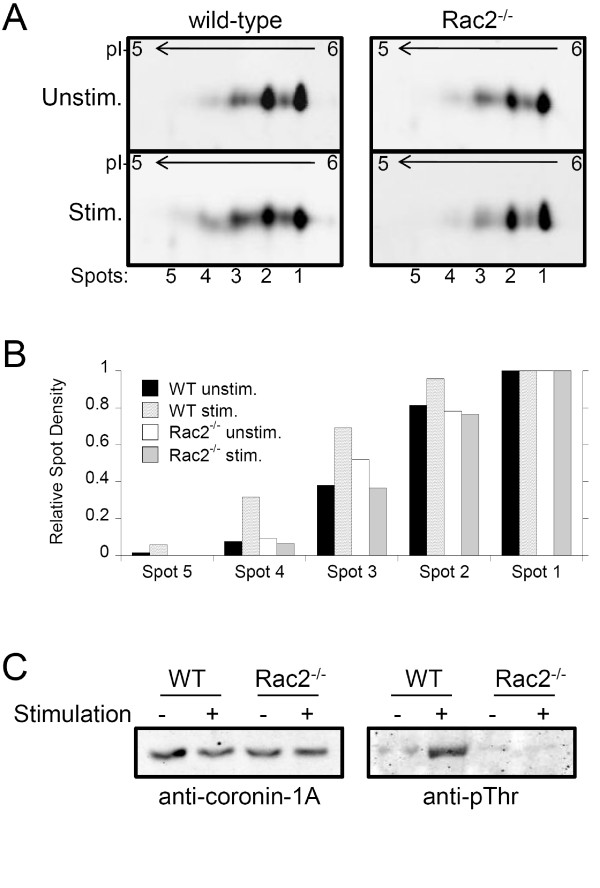
**Analysis of coronin by 2D gel electrophoresis**. Cell lysates were prepared from BMN isolated from wild-type and Rac2^-/- ^mice. Cells were pre-incubated with 10 μM cycloheximide to block *de novo *protein synthesis, then either left unstimulated (*unstim*.), or stimulated for 15 min with CB/fMLF. **A**. Lysates were subjected to 2D gel electrophoresis using non-linear pH 3 - 10 strips for isoelectrical focusing followed by 10% SDS-PAGE. Immunoblot analysis of 2D gels for coronin-1A shows multiple immuno-reactive spots that progressively migrate to more acidic pH (*spots 1 to 5*, respectively). **B**. Densiometric quantification of spots in ***A ***show increased levels of more acidic coronin-1A peptides after stimulation of BMN isolated from wild-type, but not from Rac2^-/- ^mice. **C**. Coronin-1A was immunoprecipitated from lysates and analyzed by immunoblot with phospho-specific antibodies. *Left panel *shows a 10% load control for each sample immunoblotted with anti-coronin-1A antibody. *Right panel *shows the immuno-reactivity detected with an anti-phospho-threonine specific antibody.

We next examined BMN lysates by immunoblot analysis of 1D gels to further examine changes in actin and the actin-interacting protein, coronin-1A [16]. Actin levels showed a significant reduction after stimulation of wild-type BMNs, which is consistent with the abundance changes identified in the proteomic analysis (Figure 6A, and 6C). Although coronin-1A showed a significant increase in abundance in stimulated wild-type BMNs via 2D-DiGE proteomic analysis, immunoblot analysis of 1D gels showed a less significant increase (Figure 6A, and 6B). However, immunoblot analysis of lysates run on 2D-gels revealed several coronin-1A isoforms which did not resolve by 1D electrophoresis (Figure 7A). Several coronin-1A spots in wild-type samples migrated to more acidic pI values, suggesting the appearance of various isoforms of phosphorylated coronin-1A (Figure 7, A and B, compare *wild-type unstim *to *wild-type stim*). The more acidic spots (Figure 7, spots 4 and 5) increased in abundance after stimulation, suggesting rapid multiple phosphorylation events. To test this, we immunoprecipitated coronin-1A from lysates, ran these on 1D gels, and carried out immunoblotting with anti-phospho-specific antibodies. Coronin-1A was efficiently immunoprecipitated from all lysates (Figure 7C, *left panel*), and an anti-phospho-threonine immunoreactive protein species was detected in wild-type stimulated samples (Figure 7C, *right panel*). Samples were also probed with anti-phospho-tyrosine and anti-phospho-serine antibodies, but no immunoreactive species were observed (data not shown).

## Discussion

We have previously shown that Rac2 regulates the exocytosis of primary granules which package highly reactive mediators of inflammation including myeloperoxidase and elastase [7]. However, the signaling pathway controlled by Rac2 for immune cell functions remains unclear. We took a proteomic approach to further define the molecular pathway downstream of Rac2. The application of proteomic technology has provided unique insights into both the cellular biology of neutrophil activation and the regulation of its transduction machinery [reviewed in [20]]. Proteomics approaches have been used to define identifying features of neutrophil granules and secretory vesicles [15,21,22]. In this study, we compared the protein profiles of whole-cell lysates from unstimulated and secretagogue-stimulated BMN isolated from wild-type and Rac2^-/- ^mice. Changes in protein abundance between the samples were identified, which could be derived from secretion, post-translational modification and proteolysis. Over 3500 unique spots were identified and mapped on 2D gels of fluorescently labelled protein extracts. We established peptides of interest by identifying spots that: i) increased or decreased more than 1.5-fold in abundance compared to control samples, ii) had low *p *values when comparing replicate experiments, and iii) were present in more than two-thirds of replicate experiments. With these criteria, we narrowed our search down to 22 spots to be identified by mass spectrometry; eight of these were unequivocally matched to proteins in the mouse sequence database. Changes in low abundance proteins were not detected by this method. Regulatory proteins that are in low abundance, such as GTPases, kinases and phosphatases, or integral membrane-bound proteins, such as hydrophobic transmembrane receptors, are rarely found using this approach [23].

Our findings indicated that isoforms of HSP60 and coronin were significantly increased in extracts of stimulated neutrophils, while granule proteins, β-actin, and F-actin capping protein (CapZ-β subunit) were decreased. Rac2 was required to drive the changes in the levels of these proteins upon neutrophil stimulation since little or no change was observed in neutrophil extracts from Rac2^-/- ^mice. Therefore, these differences in protein levels, which may be caused by protein modifications that increase or decrease the apparent abundance of the proteins, are likely associated with Rac2 signaling. Protein modifications that result in pI shifts are well resolved by 2D gel electrophoresis. However, immunoblot analysis for these proteins using 1D gels showed only slight changes in abundance for the primary reactive bands. This could be expected since 1D gel electrophoresis is often insensitive to changes in post-translational modifications.

Analysis of coronin-1A by 2D gel electrophoresis and immunoblot showed the presence of several isoforms. This is confirmed by previous results showing the presence of multiple phospho-isoforms of coronin-1A in macrophage lysates which resolve as a single spot after phosphatase treatment [24]. Coronin-1A and coronin-1B have been shown to be phospho-substrates of protein kinase C, which may inhibit its binding to Arp2/3 [25,26]. Recently it was shown that coronin-1A is phosphorylated on threonine-418 by cyclin-dependent kinase 5 (Cdk-5) in response to T-cell activation [27]. T-cells lacking Cdk5 do not respond to chemotactic stimuli and lack phospho-coronin. We also show coronin-1A immunoreactivity with anti-threonine antibodies after secretagogue stimulation. Therefore, Rac2 may regulate threonine phosphorylation of coronin, which may facilitate the coordination of multiple immune cell functions including polarization towards infection which is critical for both chemotaxis and degranulation.

Remodelling of actin is critical for activation of neutrophil immune functions such as chemotaxis and exocytosis and therefore we were particularly interested in the functional implications of coronin regulation. As mentioned, coronin is an actin-binding protein that is important for chemotaxis and phagocytosis [18,28], and is thought to act as a bridge between the actin cytoskeleton and the plasma membrane due to its membrane association and Arp2/3 interactions [29-31]. Interestingly, β-actin and F-actin-capping protein (CapZ-β) were also identified although these showed a decrease in abundance after stimulation of wild-type neutrophils. Barbed-end capping proteins are abundant in neutrophils, and play an important role in regulating the growth and stability of F-actin structures [19,32]. The finding that the actin capping protein, CapZ-β, is reduced supports an activation mechanism that requires actin remodelling, specifically through an uncapping mechanism, to facilitate new F-actin assembly [8,32]. The concomitant decrease in actin may be a result of transient instability during remodelling reactions. These results suggest that the actin cytoskeleton and remodelling enzymes play a regulatory role in response to secretagogue stimulation, specifically in the Rac2 signaling cascade.

Neutrophils abundantly express coronin-1A, which is associated with actin and the p40phox subunit of the NADPH oxidase complex [33]. Inhibition of coronin-1A does not affect cortical actin remodelling in macrophages [34], therefore coronin may participate in Rac-mediated polymerization of granule-associated actin for translocation to the cell cortex. Coronin is thought to act as an inhibitor of Arp2/3 in yeast [35,36]; its increase would lead to diminished actin branching which may facilitate granule docking at the cortical actin ring. A second distinct pool of actin at the cortex must be remodelled for granule docking and exocytosis. As this is the first report to show an association between Rac2 and coronin-1A, further experiments will be needed to elucidate the mechanism by which coronin-1A may be modified by the Rac2 signaling pathway.

We also identified upregulation of HSP60 as a Rac2-controlled activation mechanism in neutrophils. No connection between Rac2 and HSP60 has been previously shown. However, there is evidence that HSP60 stimulates or enhances neutrophil exocytosis and other immune cell activity [16,37,38]. It is postulated that HSP60, and perhaps other HSPs, may participate in the recruitment and activation innate and antigen presenting immune cells. Other heat shock proteins may also be required. Recently, it was shown that HSP27 regulated exocytosis in an actin-dependent manner [39]. Integration of signaling pathways with multiple chaperones is plausible given the complex mechanism required to regulate neutrophil exocytosis [6].

## Conclusion

Using whole cell proteomics, we analyzed Rac2 signaling for neutrophil function. We detected numerous changes in protein abundance in stimulated wild-type neutrophils that did not occur in cells from Rac2^-/- ^animals. Although this method was not able to detect low abundance signaling molecules, we identified several actin remodelling enzymes that had not previously been linked to downstream Rac2 signaling, including coronin-1A and HSP60. Therefore, novel signaling mechanisms that link Rac2 to actin remodelling in association with granule exocytosis have been identified using proteomic analysis. Future studies are anticipated in which the precise role of coronin-1A in neutrophil granule exocytosis will be determined.

## Methods

### Animals

Rac2 knockout (Rac2^-/-^) mice were previously generated by targeted disruption of the *rac2 *gene and were backcrossed into C57Bl/6 mice for more than 11 generations [11]. Wild-type C57Bl/6 mice were purchased from Charles River Canada (Saint-Constant, PQ). Animals were bred on site, housed under specific pathogen-free conditions and fed autoclaved food and water *ad libitum*. Mice used in these experiments were between 4-8 weeks of age. All experiments complied with the guidelines and policies of the University of Alberta's Animal Care and Use Committee and the Canadian Council on Animal Care.

### Isolation of mouse bone marrow neutrophils

Bone marrow neutrophils (BMN) were isolated from the femur and tibia of both wild-type and Rac2^-/- ^mice as previously described (5). The bones were immersed in a solution of 10X HBSS (Invitrogen, Burlington, ON) diluted to 1X with 0.1% BSA and 1% glucose (HBSS-BG), and then crushed using a mortar and pestle to liberate the bone marrow. Large debris was filtered out using a 40 μm nylon cell strainer (BD Biosciences). The filtrate was centrifuged at 300*g *for 10 min at 4°C. Percoll (GE Healthcare) stock solution was made by mixing 9:1 Percoll to 10X HBSS. Cell pellets were resuspended in 45% Percoll and then layered onto gradients consisting of 3 ml of 81%, 2 ml of 62%, 2 ml of 55% and 2 ml of 50% Percoll in HBSS-BG and centrifuged at 600*g *for 30 min at 10°C. The cell layer between 81% and 62% was harvested and washed twice in 10 ml of HBSS-BG by centrifugation at 300*g *for 10 min at 4°C. The cells were resuspended in 3 ml of HBSS-BG and were layered over 3 ml of Histopaque-1119 (Sigma-Aldrich), then centrifuged at 600*g *for 30 min at 10°C to remove contaminating erythrocytes. The cell layer between Histopaque and HBSS-BG was collected and washed twice in 10 ml HBSS-BG at 300*g *for 10 min at 4°C. The cells were resuspended in colour-free RPMI-1640 (Invitrogen) and counted by using Kimura stain (for differential counting) and Trypan Blue (for cell viability). There was an average of 3 × 10^7 ^isolated BMN per mouse. The purity of neutrophils was between 80-85% as assessed by nuclear morphology with the remainder being mononuclear cells, and viability was ≥ 90% which is similar to previously reported values [7,11].

### Analysis of stimulated BMNs

BMNs from wild-type and Rac2^-/- ^mice were separated into two conditions for proteomic analysis: unstimulated vehicle-treated controls, and cytochalasin B/formyl-Met-Leu-Phe (CB/fMLF) stimulated cells. A total of 6 × 10^6 ^cells were used for each condition. Unstimulated BMNs were treated with an equivalent level of DMSO as used for stimulated cells (0.1%), and incubated at 37°C concurrently with CB/fMLF-stimulated BMNs. For CB/fMLF treatment, cells were primed with 10 μM CB for 5 min at 37°C then stimulated to degranulate with 5 μM fMLF for 15 min at 37°C. Viability of cells was always ≥ 90% following treatment with CB/fMLF. Following stimulation, cells were centrifuged at 400*g *for 5 min at 4°. The supernatant was discarded and cell pellets were flash frozen in liquid nitrogen. BMN exocytosis was analyze by MPO assay as previously described [7] using 10^6 ^cells per ml. Where indicated, BMN were pre-incubated for10 μM cycloheximide (Sigma-Aldrich) to block *de novo *protein synthesis, which facilitates the analysis of post-translational modifications. Samples from the 0 and 20 min stimulated time points were also analyzed by microscopy for F-actin and granule distribution using rhodamine phalloidin and FITC-labelled anti-CD63 antibodies, respectively as previously described [10]. Immunoblot was performed for coronin-1A (14.1 mAb, Santa Cruz Biotechnology) and β-actin (AC15 mAb, Sigma-Aldrich). Coronin-1A was immunoprecipitated from lysates using protein-A coupled antibodies followed by immunoblot using anti-phospho-tyrosine (pY99, sc-7020 Santa Cruz Biotechnology), anti-phospho-threonine (pThr, AB1607 Chemicon) and anti-phospho-serine (pSer, AB1603 Chemicon). Densiometric quantification was performed by fluorescence detection of secondary antibodies, normalized to β-tubulin (D66 mAb, Sigma-Aldrich) using an Odyssey IR imaging system (Licor).

### Protein sample preparation and labelling with CyDye

Protein samples were prepared from unstimulated (resting) and CB/fMLF-stimulated BMN isolated from wild-type and Rac2^-/- ^mice as previously described [13]. Briefly, thawed cell pellets of 5 × 10^6 ^cells were solubilized in 0.45 ml of lysis/rehydration buffer (8 M urea, 2% (wt/v) 3-[(3-cholamidopropyl)dimethylammonio]-1-propanesulfonate hydrate (CHAPS), 1% (v/v) immobilized pH gradient (IPG) buffer (GE Healthcare) in the same pH range as the IPG strips to be used, 2 mg/ml DTT), incubated on ice for 30 min followed by a 60 s ultrasound sonication (Branson Ultrasonics, Danbury, CT) at 4°C. Samples were then centrifuged at 14,000*g *for 30 s and prepared for 2D gel electrophoresis.

To analyze the differences in protein expression between control and CB/fMLF-stimulated wild-type and Rac2^-/- ^BMN, we performed CyDye two dimensional fluorescence difference gel electrophoresis (2D-DiGE; GE Healthcare). Protein from each sample of 6 × 10^8 ^BMNs was uniquely labelled with 200 pmol of amine reactive CyDye. Approximately 0.1 mg protein was labelled with either Cy3 or Cy5 that had been freshly dissolved in anhydrous dimethyl formamide, containing 10 mM Tris-Cl, pH 8, 5 mM magnesium acetate, 8 M urea and 4% CHAPS. A pooled sample from all conditions was labelled with Cy2 and run as a normalization standard on all gels. Samples were labelled in the dark for 30 min, then terminated by adding 10 nmol lysine. An equal volume of buffer (8 M urea, 4% CHAPS, 2% (v/v) IPG buffer pH 3-10 NL and 2 mg/ml DTT) was added to each reaction. After incubation on ice for 15 min, two uniquely-labelled samples were vortexed and loaded onto one 24 cm, pH 3-10 non-linear (NL) IPG strips (GE Healthcare). A pooled sample from all conditions was labelled with Cy2 and run as a normalization standard on all gels (Figure S1).

### 2D gel electrophoresis

The samples were loaded via active rehydration at 20 V for 12 h. The strips were then focussed at 8 kV until a reading of 72 kV × h was reached. Prior to SDS-PAGE, the strips were equilibrated in 15 ml equilibration buffer (50 mM Tris-Cl, pH 8.8, 6 M urea, 30% (v/v) glycerol, 2% SDS, 10 mM DTT) for 15 min on a rocking platform. Strips were positioned on 13% SDS-PAGE gels and run at at 10°C, 2.5 W for 30 min, followed by 100 W until bromophenol blue dye front reached the gel bottom.

### Analysis of DiGE gels and protein abundance calculations

A Typhoon 9400 Imager (GE Healthcare) was used to visualize the CyDye labelled proteins. Cy2, Cy3 and Cy5 images were scanned from each gel (using lasers 488, 532 and 633 and emission filters 520BP40, 580BP30, 670BP30, respectively). Image analysis was done using the differential in-gel analysis (DIA) mode of DeCyder software (GE Healthcare).

Protein relative abundance across all samples and statistical analysis was performed using the DeCyder software biological variance analysis (BVA) module. Normalized signal intensities measured for a protein spot to the total intensity (total protein abundance) signal measured from the gel on which the protein was detected.

### Peptide selection and mass spectrometric analysis

A 2D master gels containing 0.5 mg protein was stained with Coomassie brilliant blue (PhastGel Blue R; GE Healthcare) for 12 h, then destained in 30% methanol, 10% acetic acid. Scanning was done on a flat bed computer scanner. Spot picking lists were generated in DeCyder and exported directly to the Ettan spot picker robot. Protein spots were excised from a master gel using a circular 1.5 mm picker head and put into 96-well microtitre plates.

In-gel digestion was performed using an automated Mass PREP station (Waters, Canada) according to the manufacturer's protocol. Briefly, gel plugs were washed with 50 mM ammonium bicarbonate, then 50% (v/v) acetonitrile in water. This was followed with a wash in 100% acetonitrile to dehydrate the gel plugs. The gel plugs were then reduced in 10 mM DTT and alkylated by 50 mM iodoacetamide, followed by overnight digestion with 10 μg/ml trypsin in 50 mM ammonium bicarbonate (pH 8) at 37°C. Tryptic peptides were extracted using sequential steps of 1% (v/v) formic acid, 2% (v/v) acetonitrile and 50% (v/v) acetonitrile. Liquid chromatography/mass spectrometry/mass spectrometry (LC/MS/MS) on a Micromass Q-ToF-2 mass spectrometer coupled with a Waters CapLC capillary HPLC (Waters, Canada) was used to analyse peptide extracts. Peak lists were developed using Mascot Distiller (v 1.1.1.0). Smoothing was not applied, and the peak-to-noise criterion for peak picking was 2. Centroids, or the peak's centre of mass, were calculated at peak height of 50% and charge states were calculated using the Q-ToF survey scan and peaks were de-isotoped. Protein identification from the generated MS/MS data was performed using the Mascot search engine (Mascot Daemon 2.0.1, Matrix Science). Parameters used in the search include: the enzyme was specified as trypsin; one missed cleavage was allowed; precursor mass accuracy of ± 0.6 Da; fragment ion mass accuracy of ± 0.8 Da; fixed and variable modifications were cabamidomethyl (C) and oxidation (M) respectively; and the National Center for Biotechnology Information (NCBI) non-redundant database was searched for matches. The absolute score threshold for individual peptides was examined and only those peptides with a score threshold high enough to warrant identity (>1.5 fold increase or decrease in spot abundance) were used to identify the proteins.

### Statistical analysis

Statistical comparisons were done using ANOVA with post-hoc analysis using Tukey's range test in multiple groups of samples. Comparisons were deemed significant if *p *< 0.05 and highly significant if *p *< 0.01.

## List of Abbreviations Used

BMN: bone marrow neutrophils; BVA: biological variance analysis; CB: cytochalasin B; Cy: cyanine dye; DIA: differential in-gel analysis; fMLF: formylated methionine-leucine-phenylalanine tri-peptide; LC/MS/MS: liquid chromatography tandem mass spectrometer; MS: mass spectrometer; PMA: phorbol myristate acetate; Rac2^-/-^: Rac2 gene-deletion mouse.

## Competing interests

The authors declare that they have no competing interests.

## Authors' contributions

GE conceived and coordinated the study, designed and carried out experiments, analyzed the data and wrote the manuscript. ANL handled animals, prepared samples and carried out experiments. TM handled animals, prepared samples and carried out experiments. JDK carried out experiments. DVC handled animals and prepared samples. PL conceived and coordinated the study and wrote the manuscript. All authors read and approved the manuscript.

## Supplementary Material

Additional file 1 **Figure S1 Experimental Design**. A figure outlining the protein analysis scheme, including cell isolation, protein labelling and 2D gel loading. Click here for file

Additional file 2 **Table S1. Mass spectrometry data for proteins identified in degranulation screen**. A table showing mass spectrometry data, including Mascot score, percent coverage, peptides matched and apparent molecular weight. Click here for file
