# Supplementary material for: Proteomic analysis of secretagogue-stimulated neutrophils implicates a role for actin and actin-interacting proteins in Rac2-mediated granule exocytosis
Source: Proteome Sci. 2011 Nov 14;9:70. doi: 10.1186/1477-5956-9-70 (PMC3379032; doi:10.1186/1477-5956-9-70)
Supplement: Additional file 1 — Figure S1 Experimental Design. A figure outlining the protein analysis scheme, including cell isolation, protein labelling and 2D gel loading. [file 1477-5956-9-70-S1.PDF]

## Additional File 1: Figure S1 (Eitzen et al., 2011)

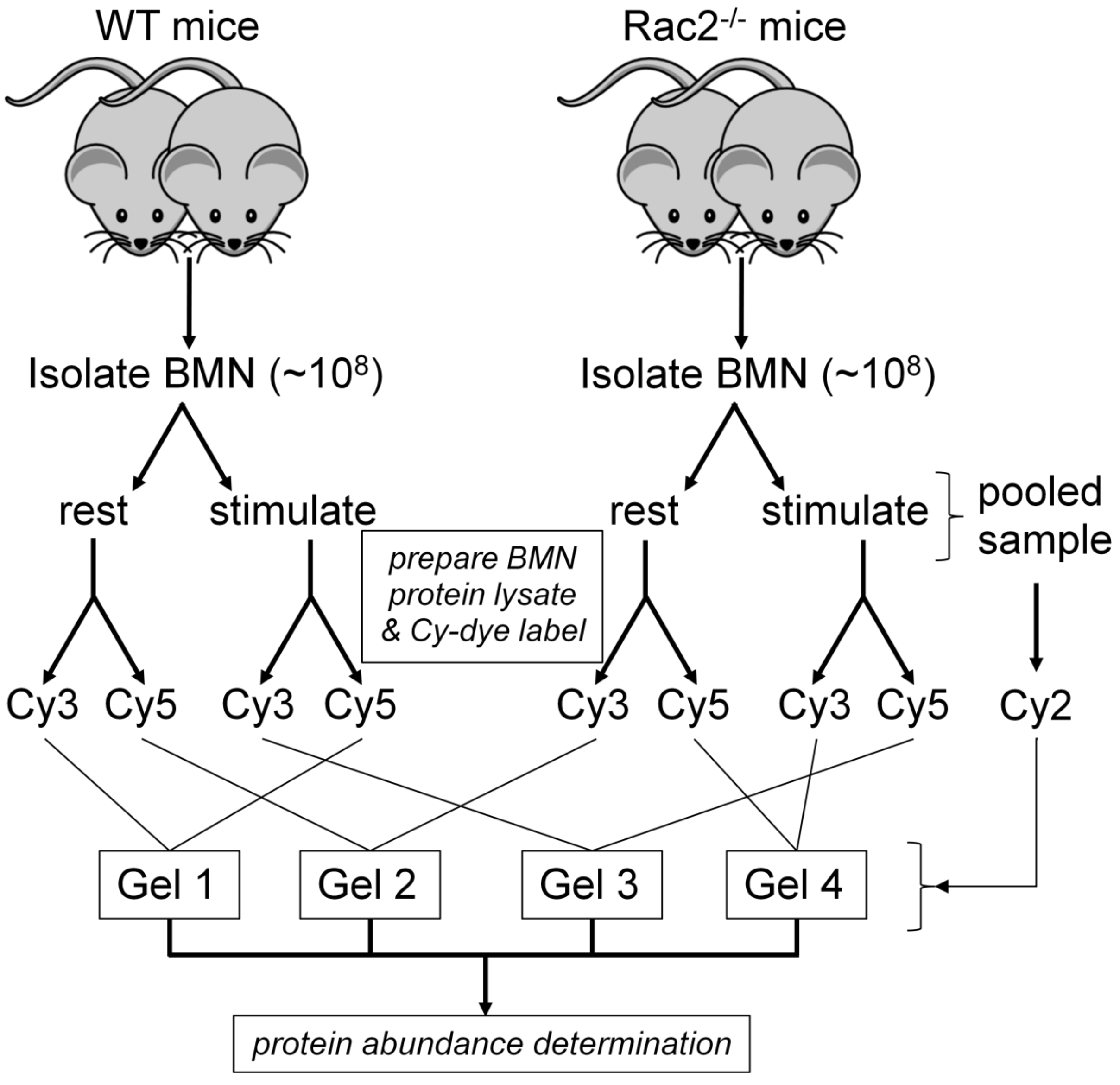


**Figure S1 Experimental Design.** BMN were harvested from the femurs of two WT or Rac2$^{-/-}$ mice, split into two samples one unstimulated (rest) and the other stimulated with CB/fMLF. After treatment proteins from each sample were labelled with Cy3 and Cy5 for a total of 8 unique samples. A pooled sample from all of the conditions was labelled with Cy2 which was used to normalize intergel variation. A Cy3 and Cy5 sample along with the pool normalization sample were run on six 2D-DiGE gels which were used for quantification of protein abundance via fluorescence scanning. The experiment was repeated three times.
