# Supplementary material for: Proteomic analysis of secretagogue-stimulated neutrophils implicates a role for actin and actin-interacting proteins in Rac2-mediated granule exocytosis
Source: Proteome Sci. 2011 Nov 14;9:70. doi: 10.1186/1477-5956-9-70 (PMC3379032; doi:10.1186/1477-5956-9-70)
Supplement: Additional file 2 — Table S1. Mass spectrometry data for proteins identified in degranulation screen. A table showing mass spectrometry data, including Mascot score, percent coverage, peptides matched and apparent molecular weight. [file 1477-5956-9-70-S2.PDF]

Additional File 2: Table S1 (Eitzen et al., 2011)

Table S1. Mass spectrometry data for proteins identified in degranulation screen

Protein	Master Number	NIH protein database accession number	Protein score [a] (Mascot v2.1)	Coverage (%)	Peptides Matched	Apparent Mwt (kDa)
Coronin	691	NP_034028	174	19.8	6	51.7
GAPDH	425	AAU89484	126	20.2	8	35.9
HSP60	382	NP_034607	97	26.1	27	61.1
Chitinase	1018	NP_075675	214	17	7	44.7
Granule protein 1	965	BAB26414	119	14.7	6	19.6
β-actin	985	ABL01512	165	27.8	15	42.1
Granule protein 2	969	NP_032720	92	13.6	4	29.7
β-actin	637	ABL01512	121	18.1	5	42.1
F-actin capping protein (CapZ-β)	999	NP_033928	190	31.4	18	31.0
Granule protein 3	970	EDL09016	71	16.2	5	29.2

[a] overall protein scores were calculated in MASCOT which reflects the confidence of matched peptides. For our analysis a cut-off score of approximately 50 reflects a 5% confidence threshold. Using this threshold, only one protein species was identified in all spots analyzed from 2D gels.
